# The Golgi protein ACBD3 facilitates Enterovirus 71 replication by interacting with 3A

**DOI:** 10.1038/srep44592

**Published:** 2017-03-17

**Authors:** Xiaobo Lei, Xia Xiao, Zhenzhen Zhang, Yijie Ma, Jianli Qi, Chao Wu, Yan Xiao, Zhuo Zhou, Bin He, Jianwei Wang

**Affiliations:** 1 MOH Key Laboratory of Systems Biology of Pathogens, Institute of Pathogen Biology, Chinese Academy of Medical Sciences & Peking Union Medical College, Beijing P.R. China; 2 Department of Microbiology and Immunology, College of Medicine, University of Illinois, Chicago, United States of America; 3 Collaborative Innovation Center for Diagnosis and Treatment of Infectious Diseases, Hanzhou 310003, Zhejiang Province, China

## Abstract

Enterovirus 71 (EV71) is a human pathogen that causes hand, foot, mouth disease and neurological complications. Although EV71, as well as other enteroviruses, initiates a remodeling of intracellular membrane for genomic replication, the regulatory mechanism remains elusive. By screening human cDNA library, we uncover that the Golgi resident protein acyl-coenzyme A binding domain-containing 3 (ACBD3) serves as a target of the 3A protein of EV71. This interaction occurs in cells expressing 3A or infected with EV71. Genetic inhibition or deletion of ACBD3 drastically impairs viral RNA replication and plaque formation. Such defects are corrected upon restoration of ACBD3. In infected cells, EV71 3A redirects ACBD3, to the replication sites. I44A or H54Y substitution in 3A interrupts the binding to ACBD3. As such, viral replication is impeded. These results reveal a mechanism of EV71 replication that involves host ACBD3 for viral replication.

Enterovirus 71 (EV71), a member of the *Picornaviridae* family, is a causative agent of the childhood exanthema known as hand, foot, and mouth disease (HFMD). In particular, infection with EV71 is often associated with neurological complications, ranging from aseptic meningitis, brainstem and cerebellar encephalitis, to acute flaccid paralysis^1^,^2^,^3^. Young children and infants are especially susceptible to EV71 infection. Since initial isolation of EV71 in the United States^4^, severe infections or outbreaks have been reported worldwide^3^,^5^,^6^,^7^. Recently, large epidemics of HFMD have occurred in the Asia-Pacific region, which raises a public health concern on EV71^5^,^7^,^8^,^9^. Currently, no specific antiviral drugs are available against EV71 infection.

EV71 is a single-stranded, positive-sense RNA virus. The viral genome is approximately 7,500 nucleotides in length, with a single open reading frame that encodes a large precursor protein. After virus infection, the precursor is processed into four structural (VP1, VP2, VP3, and VP4) proteins which are crucial for virus entry and encapsidation^10^. In addition, the precursor is cleaved into seven non-structural proteins (2A, 2B, 2C, 3A, 3B, 3C, and 3D) which mediate viral RNA transcription and translation, as well as autocatalytic polyprotein processing by 2A and 3C. The 2C, 3A and 3D proteins are required for the viral RNA replication, and are located at the RNA replication complex^11^,^12^,^13^,^14^.

Several studies suggest that enterovirus 3A plays a critical role on the formation of replication organelles^15^,^16^,^17^. It is generally believed that 3A promotes assembly of the RNA replication complex through its interaction with ARF1 or GBF1. For example, the 3A protein of poliovirus (PV) and coxsackievirus B3 (CVB3) interact with the large guanine nucleotide exchange factor GBF1^18^,^19^,^20^,^21^. However, the 3A-GBF1 interaction does not seem to correlate with RNA replication^22^,^23^,^24^. This suggests that additional factors may participate in picornavirus RNA replication. Nonetheless, the host cellular partners needed for EV71 replication are unclear.

Here we report that EV71 3A targets ACBD3, which occurs in EV71 infected cells as well. Genetic deletion of ACBD3 inhibits viral RNA replication whereas restoration of ACBD3 repairs the defect. A site-specific mutation that interrupts the 3A-ACBD3 interaction severely impairs viral replication. Further experiments reveal that GBF1 and ARF1 play a minor role on EV71 RNA replication and protein expression. Our results demonstrate that EV71 3A selectively utilizes ACBD3 to facilitate viral replication.

## Results

### ACBD3 is a cellular target of the 3A protein encoded by EV71

**Figure 1:**
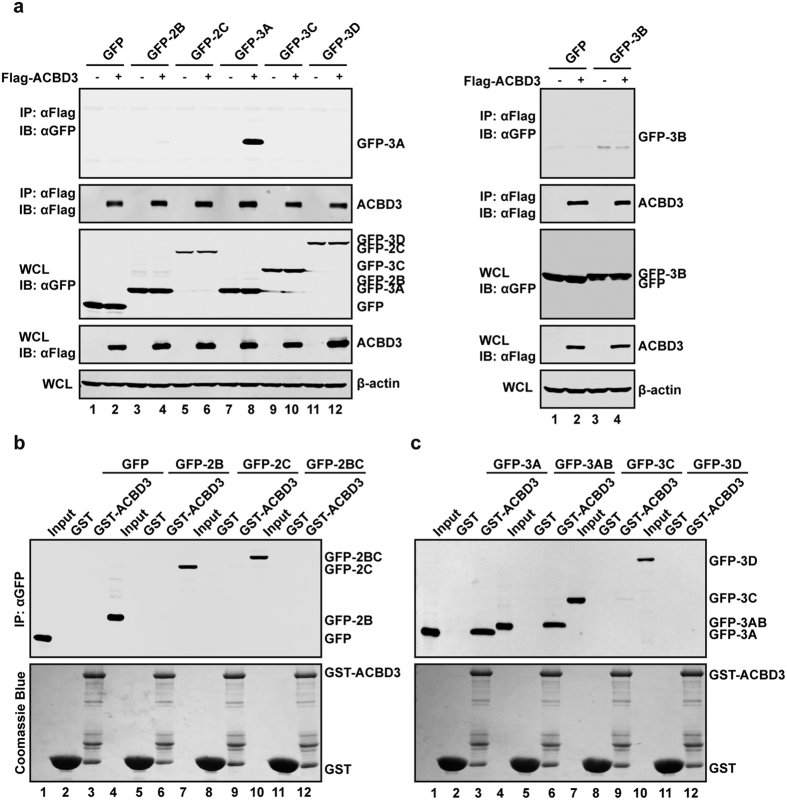
EV71 3A interacts with ACBD3. (**a**) 293 T cells were transfected with plasmids encoding Flag-ACBD3 along with GFP, GFP-2B, GFP-2C, GFP-3A, GFP-3C, GFP-3D or GFP-3B. At 24 h after transfection, cell lysates were immunoprecipitated with antibody against Flag. Samples were then subjected to Western blot analysis with antibodies against Flag, GFP and β-actin. WCL, whole-cell lysates. (b and c) 293 T cells were transfected with plasmids encoding GFP, GFP-2B, GFP-2C, GFP-2BC, GFP-3A, GFP-3AB, GFP-3C or GFP-3D. At 24 h after transfection, cells were harvested and lysated. The cell lysates were incubated with recombinant GST-ACBD3 protein which was conjugated on glutathione sepharose 4B beads at room temperature for 6 h. GST protein was used as a control. Samples were subjected to Western blot analysis with antibodies against GFP and GST. Data shown are representative of three separate experiments.

To identify host proteins that mediate EV71 genome replication, we screened human cDNA library in the yeast two-hybrid system. With 3AB, 3C and 3D of EV71 as probes, we obtained 96 positive clones. Among those, ACBD3 was identified as a 3A interacting partner. To evaluate the interaction between EV71-3A and ACBD3 in mammalian cells, 293T cells were transfected with plasmid expressing Flag-ACBD3 along with GFP-2B, 2C, 3A, 3C, 3D or 3B. GFP was used as a control. Cell lysates were then immunoprecipitated with antibody against Flag. The data in Fig. 1a show that GFP-3A was co-immunoprecipitated (Co-IP) with ACBD3, but not with 2B, 2C, 3B, 3C or 3D. To further confirm the interaction between ACBD3 with 3A, purified GST-ACBD3 was incubated with lysates of cells which express EV71 2B, 2C, 2BC, 3A, 3AB, 3C and 3D, respectively. We observed that 3A and 3AB were pulled down by GST-ACBD3, but not by GST alone (Fig. 1b and c). Taken together, these data indicate that EV71 3A specifically interacts with ACBD3.

### EV71 3A interacts with endogenous ACBD3 in virus-infected cells

**Figure 2:**
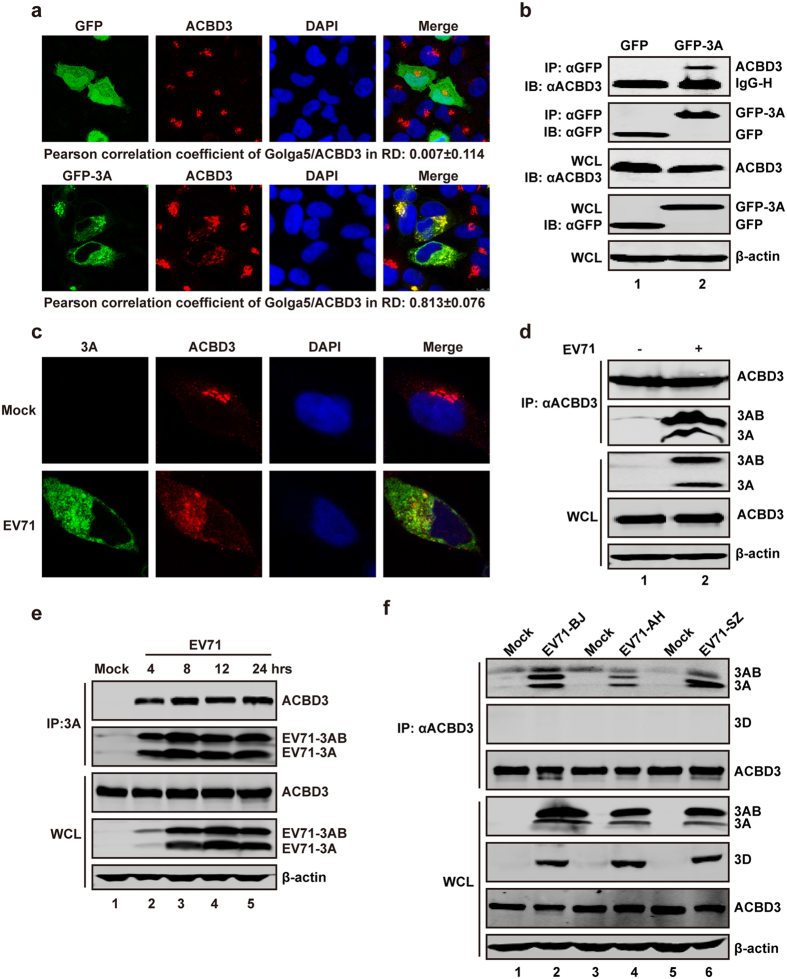
3A interacts with ACBD3 in mammalian cell. (**a**) HeLa cells were transfected with plasmids encoding GFP (Upper) or GFP-3A (Lower). At 24 h after transfection, cells were fixed and stained with anti-ACBD3 antibody (red). (**b**) 293 T cells were transfected with plasmids encoding GFP or GFP-3A. At 24 h after transfection, cell lysates were immunoprecipitated with antibody against GFP. Samples were then subjected to Western blot analysis with antibodies against ACBD3, GFP and β-actin. (**c**) RD cells were mock-infected or infected with EV71 at MOI = 5 of PFU/cell. After 8 h, cells were fixed and stained with antibodies against 3A (Green) and ACBD3 (Red). (**d**) RD cells were mock-infected or infected with EV71 at MOI = 1 of PFU/cell. After 24 h, cell lysates were immunoprecipitated with antibody against ACBD3. Samples were then subjected to Western blot analysis. (**e**) RD cells were treated as described in (**d**). At 4, 8, 12, and 24 h, cells lysates were immunoprecipitated with antibody against 3A. (**f**) RD cells were mock-infected or infected with EV71 including three different strains as indicated. After 24 h, cell lysates were immunoprecipitated with antibody against ACBD3.

To investigate whether EV71 3A interacts with endogenous ACBD3, we set out a series of experiments. As such, GFP or GFP-3A was expressed in HeLa cells. At 24 h after transfection, we examined the localization of 3A with respect to the endogenous ACBD3. In GFP-expressing cells ACBD3 primarily condensed at the Golgi apparatus (Fig. 2a, upper), which is analogous to normal cells where ACBD3 typically co-localizes with a Golgi-resident protein Golga5 (Fig. S1A). However, in GFP-3A-expressing cells ACBD3 dispersed into the cytoplasm and co-localized with 3A (Fig. 2a, lower). Consistent with this result, ACBD3 was immunoprecipitated by GFP-3A, but not by GFP (Fig. 2b). Meanwhile, the localization of endogenous ACBD3 and 3A in EV71 infected cells was determined by laser confocal microscopy. Although aggregated in the Golgi in mock infected cells, ACBD3 spread out in the cytoplasm upon EV71 infection (Fig. 2c). Notably, ACBD3 co-localized the 3A protein of EV71 in discrete regions (Fig. 2c). Immunoprecipitation analysis shows that 3A and 3AB was pelleted by anti-ACBD3 antibody in EV71 infected cells (Fig. 2d). Western blot analysis verified the expression of ACBD3 both in mock and virus infected cells (Fig. 2d). Furthermore, time course analysis reveals that ACBD3 was pelleted by anti-3A antibody as early as 4 h after EV71 infection (Fig. 2e). These results suggest that 3A interacts with and redirects ACBD3 to distinct regions in EV71 infected cells.

To further test the 3A-ACBD3 interaction, we assessed EV71 strains from Beijing (BJ), Anhui (AH), and ShenZhen (SZ) of China. In immunoprecipitation assay, the 3A proteins from these strains were immunoprecipiated by anti-ACBD3 antibody after virus infection (Fig. 2f). Furthermore, we detected the interaction of three 3A proteins with ACBD3 by immunofluorescence staining. The results indicate that at 4 h post infection, ACBD3 dispersed to cytoplasm and partially localized with 3A (Fig. S1B). As infection continued, a notable fraction of ACBD3 co-localized with 3A (Fig. S1C). We conclude that the 3A protein mediated translocation of ACBD3 is not limited to a specific EV71 strain.

### ACBD3 is necessary for replication of EV71

**Figure 3:**
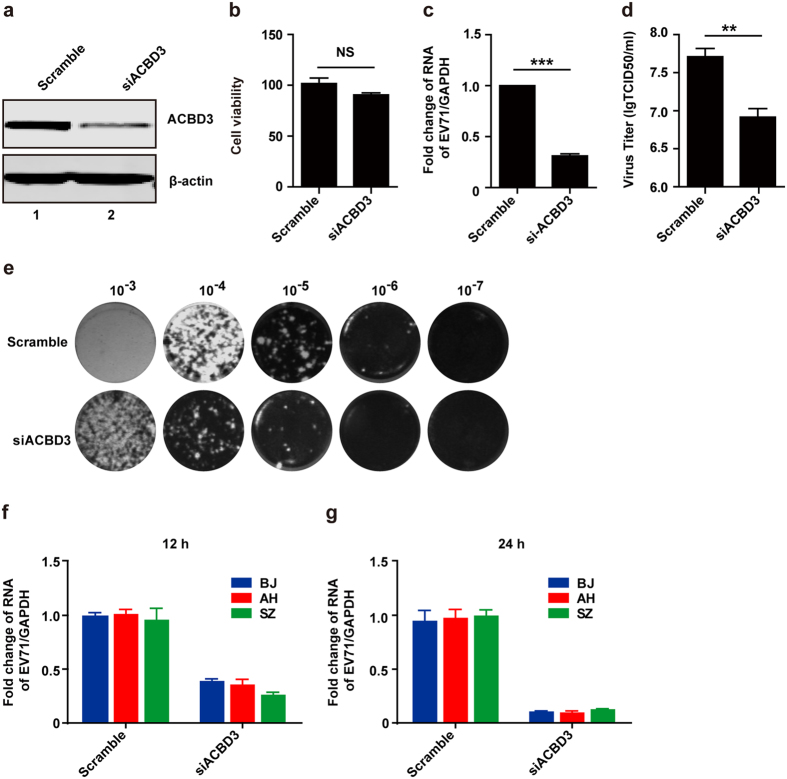
ACBD3 is necessary for EV71 replication. (**a**) RD cells were transfected with siRNA against ACBD3. At 48 h, cells were harvested for Western blot analysis. (**b**) RD cells were treated as indicated in (**a**). Cell viability was detected by using Cytotoxicity Detection Kit^Plus^ (LDH). (**c**) RD cells were transfected with siRNA against ACBD3. After 48 h, cells were infected with EV71 at MOI of 1 PFU/cell. At 24 h, total RNA was extracted and the viral RNA levels of EV71 were evaluated by quantitative real-time PCR using SYBR Green. Data are expressed as fold change of the EV71 RNA level relative to the control using the ΔΔCT method as described in the Materials and Methods. (**d**) RD cells were treated as indicated in (**c**). Cells were harvested for tissue culture infective dose (TCID50) assay. (**e**) Plaque assay of viruses were performed on RD cells at 37 °C. Monolayers were stained with crystal violet at 72 h post infection. Data shown are representative of three independent experiments. (**f** and **g**) RD cells were transfected with siRNA against ACBD3. At 48 h, cells were infected with different EV71 strains as indicated. After 12 (**f**) or 24 (**g**) h, total RNA was extracted and the viral RNA levels of EV71 were evaluated by quantitative real-time PCR using SYBR Green.

Based on the above results, we hypothesized that EV71 3A mediates viral replication through its interaction with ACBD3. To test this, we performed siRNA knockdown assays. RD cells were transfected with small interfering RNA (siRNA) against ACBD3 or a control. As expected, ACBD3 expression was significantly reduced in cells transfected with ACBD3 specific siRNA but not in cells with scramble siRNA (Fig. 3a). This reduction had no apparent cytotoxicity as measured by cell viability assay (Fig. 3b). Next, we determined the effect of ACBD3 on EV71 replication. Cells were infected with EV71 at 48 h after transfection with siRNA targeting ACBD3. Total RNAs were prepared at 24 h post infection and subjected to RT-PCR analysis. As shown in Fig. 3c, siRNA knockdown of ACBD3 resulted in 70% decrease in the level of EV71 RNA compared to the control, indicating an inhibition of viral replication. In line with this, knockdown of ACBD3 reduced viral yield (Fig. 3d). As corroborative approach, we carried out plaque assay. Figure 3e shows that ACBD3 knockdown sharply reduced plaque formation. Moreover, these phenotypes were seen with different EV71 strains (Fig. 3f and g).

**Figure 4:**
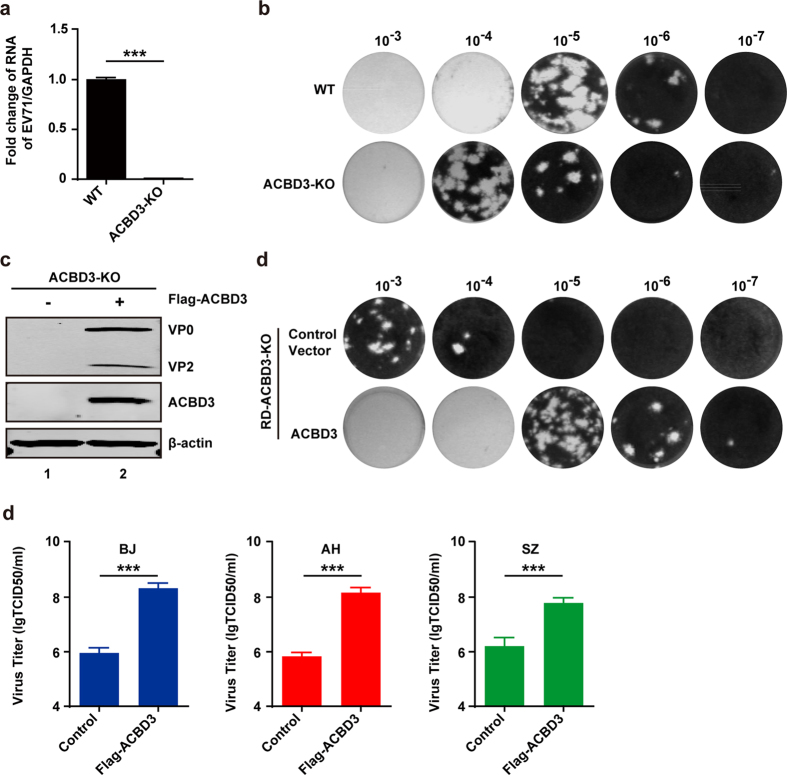
The expression of ACBD3 rescues EV71 replication. (**a**) The effect of *ACBD3*^−/−^ on EV71 replication by RT-PCR assay (**a**) or plaque assay (**b**). Values represent the mean of triplicate samples ± SEM. Error bars represent SEM. (**c** and **d**) The plasmid which expresses ACBD3 was transfected into the ACBD3 knockout cells. After 24 h, cells were infected with EV71. At 24 h, the replication of EV71 was detected by WB (**c**) or plaque assay (**d**). (**e**) The plasmid which expresses ACBD3 was transfected into the knockout cells. After 24 h, cells were infected with different EV71 strains respectively. At 24 h, the replication of EV71 was detected by TCID50. Values represent the mean of triplicate samples ± SEM. Error bars represent SEM.

To define the role of ACBD3 unambiguously, we generated an *ACBD3*^−/−^cell line by CRISPR-mediated disruption (Fig. S2A and S2B). As illustrated in Fig. 4a, viral RNA replication was readily detected in wild type cells. Nevertheless, it was severely impeded in *ACBD3*^−/−^ cells. This was not due to a defect in viral entry (Fig. S2C). Congruently, the efficiency of plaque formation was drastically reduced in the absence of ACBD3 (Fig. 4b). In addition, viral protein synthesis was barely visible in *ACBD3*^−/−^ cells (Fig. 4c). When ectopically expressed, ACBD3 restored viral protein synthesis and the efficient of plaque formation (Fig. 4c and d). Similarly, addition of ACBD3 restored viral growth in *ACBD3*^−/−^ cells infected with different EV71 strains (Fig. 4e). These experimental results demonstrate that ACBD3 is indispensable for EV71 replication.

### Isoleucine 44 and histidine 54 of 3A are functionally important

**Figure 5:**
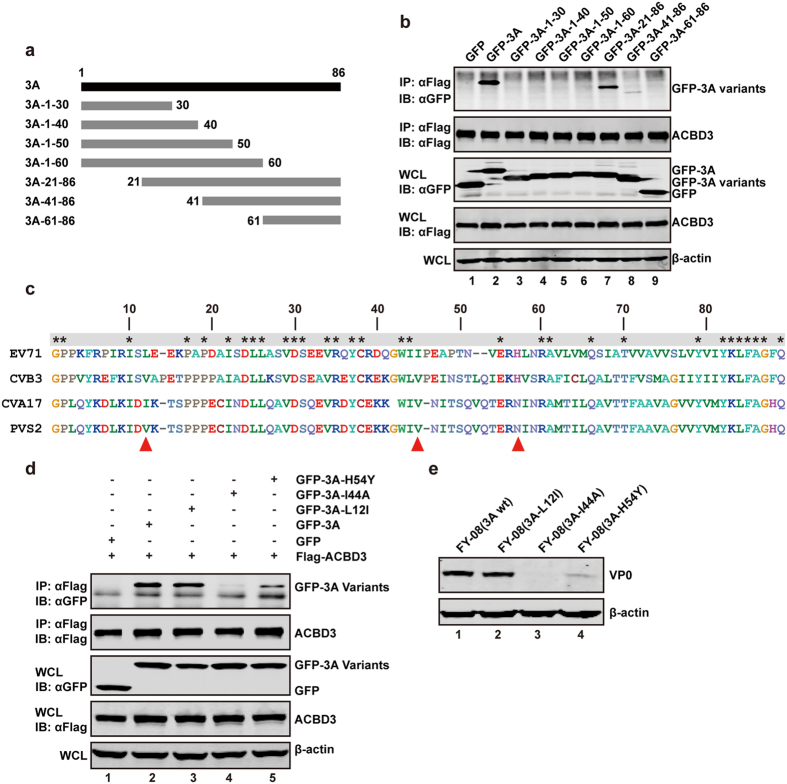
ACBD3 interacts with 3A at C-terminal amino acids. (**a**) Schematic diagram of different variants of 3A. (**b**) 293 T cells were transfected with plasmids encoding GFP or GFP-3A variants along with Flag-ACBD3. At 24 h after transfection, lysates were immunoprecipitated using anti-Flag. Samples were subjected to Western blot analysis. (**c**) Amino acids sequence alignment of 3A protein of EV71, CVB3, CVA17 and PVS2. (**d**) 293 T cells were transfected with plasmids encoding GFP or GFP 3A amino acid substitution mutants along with Flag-ACBD3, the immunoprecipitation were done as described above. (**e**) RD cells were transfected with EV71 RNA transcripts. At 72 h post transfection, cells were harvested and resolved with 12% SDS-PAGE. Western blot analysis for EV71 or β-actin was conducted.

Given that 3A interacted with ACBD3, we sought to define the interaction domains. First, we mapped the 3A interacting domain of ACBD3 to the C-terminal GOLD (Golgi dynamic) region (Fig. S3). Next, we investigated region of 3A protein responsible for the ACBD3 interaction. As EV71 3A is a small protein, of 86 amino acids, we constructed a panel of plasmids expressing 3A mutants (Fig. 5a). These include the four N-terminal fragments (1–30, 1–40, 1–50, 1–60aa) and three C-terminal fragments (21–86, 41–86, 61–86aa). 293 T cells were transfected with flag-ACBD3 along with mutants of GFP-3A or GFP. Cell lysates were prepared for Co-IP assays using anti-flag antibody. As shown in Fig. 5b, the full length 3A was co-precipitated with ACBD3. Similarly, the 21–86 mutant was also co-precipitated with ACBD3, indicating that amino acids 1–21 is not critical. However, the 41–86 mutant only weakly bound to ACBD3, which suggests a requirement of this region. The 3A mutants with deletions of amino acids 30–86 were unable to bind ACBD3. These data suggest that at least the region of 3A encompassing amino acids 41–86 is required to bind ACBD3. In additional, we performed secondary structure analysis via Phyre2 computer protein analysis program. It seems EV71 3A may potentially form 3α-helix (amino acids 19–28, 31–39, and 48–60). While H54 falls in the third a-helix, I44 sits in a random coiled region. A relevant structure is not apparently evident. Additional work is needed to elucidate the structure-function relationship.

Alignment of amino acid sequences shows that 3A from enteroviruses is homologous but with notable variations (Fig. 5c). It has been reported that isoleucine 12 in 3A of CVA17 is critical for binding to GBF1^19^, but plays no role in binding to ACBD3^25^. And valine 44 and histidine 57 of 3A from CVB3 are functionally linked to PI4KB in viral replication^26^,^27^. To define the role of these residues in EV71 replication, we constructed mutants of EV71 3A, with L12I, I44A, or H54Y substitution. To evaluate the 3A mutations on ACBD3 binding, 293 T cells were transfected with ACBD3 along with 3A variants and subjected to immunoprecipitation. As illustrated in Fig. 5d, like wild type 3A, L12I was able to bind ACBD3 (lanes 2 and 3), suggesting leucine 12 is not required. Although expressed at comparable levels, I44A lost its ability to bind ACBD3 (lane 4) whereas H54Y bound ACBD3 weakly (lane 5). These results argue that isoleucine 44 and histidine 54 dictate the interaction of EV71 3A and ACBD3.

To assess 3A mutations on viral replication, we generated recombinant EV71. RD cells were infected with 3A variants and lysates of cells were processed for Western blot analysis with anti-VP0. As shown in Fig. 5e, similar to wild type virus, the 3A-L12I mutant expressed VP0, suggesting normal viral replication (lanes 1 and 2). In sharp contrast, 3A-I44A and 3A-H54Y barely produced any VP0 (lane 3 and 4). Herein, either I44A or H54Y substitution was important to EV71 replication. These results suggested that 3A-ACBD3 interaction is critically important for viral replication.

### GBF1 and ARF1 have a minor effect on viral genome replication

**Figure 6:**
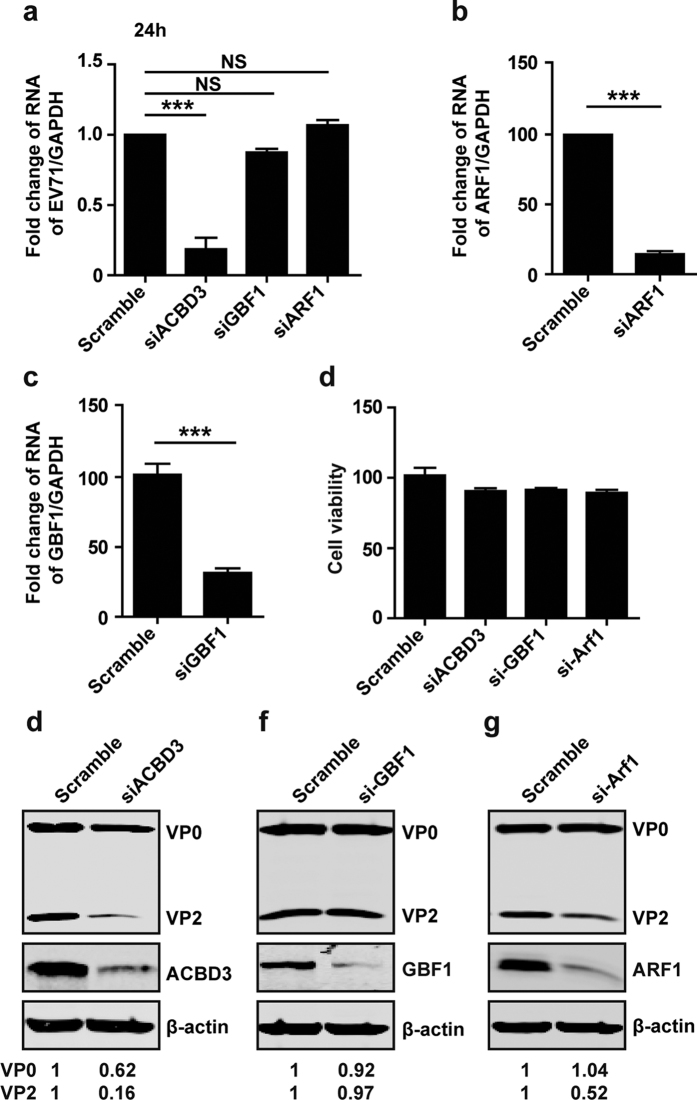
GBF1 and ARF1 have a minor effect on viral RNA replication and protein expression. (**a**) RD cells were transfected with siRNA against ACBD3, GBF1 and ARF1. At 48 h, cells were infected with EV71 at MOI = 1 of PFU/cell. After 24 h, total RNA was extracted and the viral RNA levels of EV71 were evaluated by quantitative real-time PCR using SYBR Green. (b and c) The knockdown efficacy of si-ARF1 (**b**) or si-GBF1 (**c**) was detected by RT-PCR assay at 48 h post-transfection in RD cells. Data shown are representative of three independent experiments. (**d**) Cell viability was detected by using Cytotoxicity Detection KitPlus (LDH). (**e**–**g**) RD cells were transfected with siRNA against ACBD3 (**e**), GBF1 (**f**), or ARF1 (**g**). At 48 h, cells were infected with EV71 at MOI of 1 PFU /cell. At 24 h, cells were harvested for Western blot assay.

The formation of replication organelles of enteroviruses is dependent on GBF1 and ARF1^13^,^18^,^20^,^28^. To investigate whether EV71 replication relies on GBF1or ARF1, we analyzed viral RNA and proteins. As shown in Fig. 6a, depletion of ACBD3 by siRNA severely impeded viral replication, with 75% of reduction in viral RNA synthesis. At 24 h post-infection, depletion of GBF1 exerted partial inhibition viral RNA replication (Fig. 6a), although siRNA specific to ARF1 or GBF1 was functionally active (Fig. 6b and c). Under these experimental conditions, siRNA treatment was not toxic to the cells (Fig. 6d). Depletion of ACBD3 sharply reduced VP2 production (Fig. 6e). However, Depletion of ARF1 or GBF1 marginally inhibited VP2 production (Fig. 6f and g).

**Figure 7:**
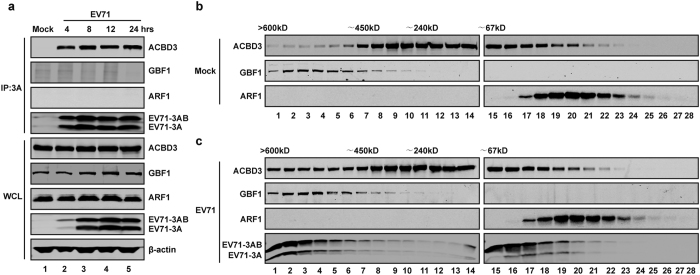
GBF1 and ARF1 do not localize in the replication organelles. (**a**) RD cells were mock-infected or infected with EV71 at MOI = 1 of PFU/cell. At 4, 8, 12, and 24 h, cell lysates were immunoprecipitated with antibody against 3A. Samples were detected by WB assay using indicated antibodies. (**b** and **c**) RD cells were mock infected (**b**) or infected with EV71 (**c**) for 12 h. Cell lysates were fractionated on a size-exclusion column using Superdex 200 10/300 GL. Each fraction was analyzed by Western blot using the indicated antibodies. The positions corresponding to the elution of the standard markers of molecular weight are indicated.

Next, we asked whether ARF1 or GBF1 interacted with viral replication machinery. Immunofluorescence analysis shows that GBF1 spread out as speckles in mock infected cells (Fig. S4a). At 4, 6, and 8 hours after infection, the localization coefficient between GBF1 and 3A are less than 0.35. Similarly, the localization coefficient between ARF1 and 3A are less than 0.3 at 4, 6, and 8 hrs post-infection (Fig. S4b). EV71 infection induced their accumulation at 12 hrs post-infection (Fig. S4a and S4b). To further corroborate this, we carried out immunoprecipitation analysis. RD cells were mock infected or infected with EV71. At different time points post infection, lysates of cells were subjected to immunoprecipitation with anti-3A antibody. Although ACBD3, EV71 3A and 3AB were precipitated with 3A neither GBF1 nor ARF1 was associated with 3A in virus infected cells (Fig. 7a). This coincided with the fractional analysis by size-exclusion chromatography assay. The elution peaks of GBF1 and ARF1 has not changed after EV71 infection compared to mock infection (Fig. 7b and c). Taken in combination, these data suggest that ARF1 and GBF1 have a minor role in the replication EV71.

## Discussions

Enterovirus 71 is a human pathogen that causes hand, foot, and mouse disease and neurological complications. Although members of enteroviruses are extensively investigated, the mechanism of viral replication is unclear. Here we demonstrate that the 3A protein of EV71 mediates viral replication through host ACBD3. ACBD3 is crucial in Aichi virus replication, yet its relation with enteroviruses is a puzzle^29^,^30^. A number of studies showed that ACBD3 physically interacts with 3A encoded by enteroviruses, but is dispensable in viral replication^13^,^23^,^24^,^25^,^31^. This is evident in cells infected with rhinovirus, poliovirus and coxsakievirus B3. The results present in this work uncovered that EV71 3A associated with and directed ACBD3 to discrete regions in the cytoplasm. This is different from a report that no interaction between EV71 and ACBD3 was detectable^30^. The basis for this discrepancy is not known. Our work suggests that is probably a prerequisite to initiate sites or platforms necessary for RNA replication. Consistent with this interpretation, depletion or knockout of ACBD3 precluded viral RNA replication, protein synthesis and plaque formation. Furthermore, these phenotypes were also noted with several different strains of EV71. Strikingly, a site-specific mutation in 3A of EV71 disrupted the 3A-ACBD3 interaction and viral replication. Therefore, besides an ACBD3 binding site an additional cis-element in 3A may be required. Collectively, these results support the argument that engagement of EV71 3A with ACBD3 dictates viral replication. It remains obscure why ACBD3 is differentially involved in viral replication. As 3A from picornaviruses exhibits only about 42% homology, amino acid sequence variations may have a role. An attractive hypothesis is that 3A may bear distinct features that require or bypass ACBD3. Another possibility is that a separate viral protein may substitute for the function of ACBD3.

Enterovirus replication is reported to involve ARF1 and GBF1^11^,^32^. In this model, membrane-bound 3A modulates ARF1/GBF1 to recruit PI4KB, resulting in a replication complex. Intriguingly, knockdown of ARF1 or GBF1 had a minor inhibitory effect on EV71 replication. However, depletion of ARF1 had a marginal effect on VP2 production. Further, in cells infected with EV71 ARF1/GBF1 was not recruited to form replication organelles. While 3A/3AB formed complex with ACBD3, no ARF1/GBF1 was detectable. These results are consistent with the notion that EV71 replication only partially dependent on ARF1 or GBF1. In line with this, GBF1 and ARF1/ARF3 was necessary for EV71 replication at 6 hrs^32^, suggesting that GBF1 and ARF1/ARF3 may play a role in the early stage of viral replication. It seems that EV71 has evolved an alternative mechanism to hijack ACBD3, which substitutes for ARF1/GBF1. In this regard, it is notable that depletion of ACBD3, GBF1 and ARF1 does not affect the formation of replication complex in coxsakievirus B3 infection^24^. It remains an open question why distinct mechanisms are operative within enteroviruses.

In summary, enteroviruses remodel cellular membranes into specialized structures where viral replication complexes are assembled coordinately. This study highlights an essential role of ACBD3 in the replication of EV71. This is probably relevant to the biology different picornaviruses. It is likely that the ACBD3 pathway is not only important in Aichi virus but also in EV71 replication.

## Methods

### Cell lines and viruses

Human rhabdomyosarcoma (RD) cells, HeLa cells, and human embryonic kidney HEK-293T cells, were cultured in Dulbecco’s modified Eagle’s medium (DMEM, Gibco) supplemented with 10% heated-inactivated fetal bovine serum (FBS) (Hyclone, Logan, UT), 100 U/ml penicillin and 100 μg/ml streptomycin. All the cells were cultured at 37 °C in a 5% CO_2_ humidified atmosphere. EV71 infection was carried out as described previously^33^,^34^,^35^. The EV71 strain SHZH98 (GeneBank Accession no. AF302996.1), Anhui Fuyang-0805 (GeneBank accession no. F439769) and BJ/CHN/2008 (GeneBank accession no. HQ615421.1) were used in this study.

### Plasmids

The plasmids pEGFP-C1, pEGFP-2B, pEGFP-2C, pEGFP-3A, pEGFP-3AB, pEGFP-3C, and pEGFP-3D have been described elsewhere^35^. The plasmid expressing human ACBD3 was purchased from Origene (Rockville MD). The plasmids expressing GST-fused ACBD3 variants are gifts from Zhang Leiliang (Institute of Pathogen Biology, Chinese Academy of Medical Sciences, China)^36^. The ACBD3 and pEGFP-3A variants were constructed by site-directed mutagenesis using Pfu DNA polymerase (Strategene, La Jolla, CA). The primers for 3A variants were used as indicated in supplementary Table 1. All variants were verified by nucleotide sequence analysis. LentiCRISPRv2, pVSVg, psPAX2 were obtained from AddGene.

### Antibodies and reagents

Antibodies against Flag, GFP, Myc and β-actin were purchased from Sigma (St. Louis, MO). Anti-ACBD3 was purchased from Santa Cruz Biotechnology (Santa Cruz, CA) and Sigma (St. Louis, MO). Mouse anti-enterovirus 71 was purchased from Chemicon (Billerica, MA). Polyclonal antibody against GBF1 was obtained from Abcam (Cambridgy, UK) and Proteintech (Rosemont, IL). The monoclonal antibody against ARF1 was obtained from Novus (Charles, MO) and Proteintech. The Rabbit anti-Golga 5 was purchased from Sigma. Primary rabbit or mouse EV71 3A antibody was generated against a peptide MGPPKFRPIRISLEEKPAPDC. Goat-anti mouse or rabbit secondary antibodies were purchased from Li-COR Company (LI-COR Inc., Lincoln, NE). Alexa Flour 488-conjugated anti-mouse IgG and anti-rabbit IgG antibodies, Alexa Flour 549-conjugated anti-mouse IgG anti-mouse IgM, anti-rabbit IgG were purchased from Molecular probes (Eugene, OR, USA).

### Protein purification and GST pull down assay

The expression and purification of GST fusion proteins were carried out according to Amersham handbook. GST pull down assays were performed as previously described method^37^. Briefly, 2 μg purified GST-ACBD3 variants or GST were incubated with 100 μg lysates of cells which expressing the EV71 2B, 2C, 3A, 3AB, 3C, or 3D at 4 °C for 6 h in binding buffer, respectively. Then 20 μl of 50% slurry glutathione-Sepharose 4B was added to the reaction mixture which was adjusted to 200 μl with binding buffer (20 Mm Tris-HCl, 150 mM NaCl, pH7.4), rotated on a wheel at 4 °C for 2 h. These mixtures were washed four times with washing buffer. The samples were analysis by Western blot assays.

### siRNA mediated gene silencing in RD and HeLa cells

RD or HeLa cells were transfected with 40 nM of small interfering RNA (siRNA) oligos for 48 h using DharmaFECT1 (Dharmacon). siRNA target sequences are shown below (forward): ACBD3: 5′-GGAUGCAGAUUCCGUGAUU-3′; GBF1: 5′-CAGGAGCATGTACATATGGAA-3′; ARF1: 5′-ACGATCCTCTACAAGCTTA-3′.

### Generation of CRISPR-Cas9 knockout cell lines

The target sequence used is TCGCCACCTGGATCCGGTCG for human ACBD3. First, the sequences were cloned into lenti-CRISPRv2 which was digested by BsmBI. To construct the knockout cell lines, 1.2 μg of gRNA-expressing plasmid, 0.6 μg pVSVg plasmid and 0.9 μg of psPAX2 vector were co-transfected into1.2 × 10^6^ HEK293T cells. At 48 hrs post-transfection, the supernatant of transfected cells which contain lentivirus were collected for the following experiment.

RD cells were plated into 24-well culture plate. The next day, lentivirus was added to the cells. After 48 h, cells infected by lentivirus were screened by using 1 μg/ml puromycin. Five days later, single clones were screened by limiting dilution cloning method. The knockout clones were verified by sequencing of the PCR fragments and Western blotting assay. The PCR primers are: 5′-GGCAACTGACGATTCCGGAGGG-3′ and 5′-CGGGACATGACTGCTAAACCAA-3′.

### Reverse transcription-PCR

Total RNA was extracted from cells by using TRIzol reagent (Invitrogen, Carlsbad, CA). RNA samples were treated with DNase I (Pierce, Rockford, IL) and reverse transcription was carried out using the Superscript cDNA synthesis kit (Invitrogen) according to the manufacturer’s instructions. cDNA samples were subjected to real-time PCR by using SYBR Green. The primers used were as follows: GBF1 (5′-GGGAACGCATTGACTGTTTT-3′ and 5′-CTCGGGCTTCTCAAAGTCAC-3′), ARF1 (5′-GTGACCACCATTCCCACCATAG-3′ and 5′-TCATTGCTGTCCACCACGAAG-3′), GAPDH (5′-CAACTGGTCGTGGACAACCAT-3′ and R 5′-GCACGGACACTCACAATGTTC-3′). Levels of genes mRNA were normalized to GAPDH mRNA. Results are reported as fold change using the ΔΔCT method.

### Plaque assay

RD cells (2 × 10^5^) were seeded in 24-well plate with growth medium (DMED, 10% FBS). The next day, growth medium were removed and 200 μl serial dilution of EV71 stocks were added to well. Then the 24-well plates were incubated at 37 °C for 2 h. 1 ml per well 1.2% Avicel (R-591, FMC) (one volume 2.4% avicel with the same volume 2 × DMEM) were added to the plate, as previously described^38^. After72 h, cells were fixed and stained with crystal violet.

### Western blot analysis

Cells were washed and lysed in RIPA buffer containing 150 mM NaCl, 25 mM Tris pH 7.4, 1% NP-40, 0.25% sodium deoxycholate, 1 mM EDTA with protease inhibitor cocktail (Roche, Indianapolis, IN). The lysates were centrifuged at 16,000 g for 10 min at 4 °C. Aliquots of cell lysates were electrophoresed on SDS-PAGE gels and were transferred to a nitrocellulose membrane (Pall, Port Washington, NY). The membranes were blocked with 5% non-fat dry milk and then proteins on the membrane were incubated with indicated primary antibodies at 4 °C overnight. This was followed by incubation with corresponding IRD Fluor 800-labeled IgG or IRD Fluor 680-labeled IgG secondary antibody (LI-COR Inc., Lincoln, NE) for 1 h at room temperature. After washing, the membranes were scanned with the Odyssey Infrared Imaging System (LI-COR, Lincoln, NE) at a wavelength of 700–800 nm and the molecular sizes of the developed proteins were determined by comparison with pre-stained protein markers (Ferments, Maryland). Western blot band densities were quantitated by the Image J software.

### Immunoprecipitation

Transfected cells were lysed with RIPA, containing protease inhibitor cocktail (Roche, Indianapolis, IN). Lysates of cells were incubated with primary antibodies in 500 μl RIPA buffer at 4 ^o^C overnight on a rotator in the presence of protein A/G agarose beads (Santa Cruz Biotechnology, Santa Cruz, CA). Immunocomplexes captured on the protein A/G agarose were fractionated by 8–15% SDS-PAGE and transferred to nitrocellulose membranes for analysis.

### Immunofluorescence assay and Confocal Microscopy

Immunofluorescent assay was done as described previously^39^. Briefly, cells were fixed with 4% formalin followed by washing with PBS buffer, and permeabilized with 0.5% Triton X-100. Then cells were blocked and stained with primary antibodies, followed by stained with an Alexa Fluor 488/594 secondary antibodies. Nuclei were counterstained with DAPI (Sigma). Fluorescence images were obtained and analyzed using a laser scanning confocal microscope (Leica TCS SP5). For correlation analysis, the Pearson correlation coefficient values were calculated using the WCIF Image J software with colocalization threshold plugin.

### Size-Exclusion Chromatography (SEC) by using Superdex200 10/300 GL

6 × 10^7^ RD cells were mock-infected or infected with EV71 for 12 h. Cells were harvested and lysed in buffer comprised of 14 mM CHAPS, 150 mMNaCl, and 20 mMTris-HCl (pH7.4). Cell lysates filtered with 0.2 μm membrane filters were loaded onto a Superose 200 10/300 GL column (GE Healthcare, Life Sciences). Elution was performed at 4 °C and monitored by absorbance at 280 and 260 nm. Every 0.5 mL of fraction was collected. Each fraction was analyzed by Western blotting assay. Molecular weight was determined using standard proteins (Ferments, Maryland).

### EV71 and mutated-EV71 infectious clones

The EV71 infectious clone was done as described in refs 40 and 41. The mutated infectious clones were constructed by site-directed mutagenesis using Pfu DNA polymerase (Strategene, La, Jolla, CA) using the wild type EV71 infectious clone as a template. RNA transcripts were synthesized *in vitro* from linearized templates using MEGAscript^®^ T7 Kit (Invitrogen). Then, RNA transcripts were transfected into RD cells. At 72 h post-transfection, viruses were collected and analyzed by Western blot using an anti-EV71 antibody.

### Statistics

The two-tailed Student’s *t-*test was used for two-group comparisons. The values P < 0.05 (*), P < 0.01(**) and P < 0.001(***) were considered significant. NS stands for not significant.

## Additional Information

**How to cite this article**: Lei, X. *et al*. The Golgi protein ACBD3 facilitates Enterovirus 71 replication by interacting with 3A. *Sci. Rep.* **7**, 44592; doi: 10.1038/srep44592 (2017).

**Publisher's note:** Springer Nature remains neutral with regard to jurisdictional claims in published maps and institutional affiliations.

## Supplementary Material

Supplementary Information
