# Supplementary material for: The Golgi protein ACBD3 facilitates Enterovirus 71 replication by interacting with 3A
Source: Sci Rep. 2017 Mar 17;7:44592. doi: 10.1038/srep44592 (PMC5356004; doi:10.1038/srep44592)

The Golgi protein ACBD3 facilitates Enterovirus 71 replication by interacting with 3A

Xiaobo Lei[1]#, Xia Xiao[1, 2]#, Zhenzhen Zhang[1], YijieMa[3], Jianli Qi[1], ChaoWu[1], Yan Xiao[1], Zhuo Zhou[1], Bin He[3]*, Jianwei Wang[1, 2]*

[1]MOH Key Laboratory of Systems Biology of Pathogens, Institute of Pathogen Biology, Chinese Academy of Medical Sciences & Peking Union Medical College, Beijing P.R. China
[2]Collaborative Innovation Center for Diagnosis and Treatment of Infectious Diseases, Hanzhou 310003, Zhejiang Province, China
[3]Department of Microbiology and Immunology, College of Medicine, University of Illinois, Chicago, United States of America

#These authors contributed equally to this work.

*Correspondence and requests for materials should be addressed to J.W. (email: wangjw28@163.com) or B.H. (email: tshuo@uic.edu)

Running title: ACBD3 is necessary for EV71 replication

Supplementary information

Supplemental Figure Legends

Supplementary Figure S1. The localization of ACBD3 and 3A. (a) ACBD3 located at Golgi. RD cells were fixed and labeled with anti-Golga5 and ACBD3 antibodies. Images were obtained using confocal microscopy. (b and c) RD cells were mock-infected or infected with EV71 including three different strains, BJ, AH, and SZ strains. After 4 (b) or 8 (c) h, cells were fixed and labeled with anti-3A (Green) and ACBD3 (Red) antibodies.

Supplementary Figure S2. Generation of ACBD3$^{-/-}$ cell lines and the effect of ACBD3 on viral entry. (a) Generation of *ACBD3*$^{-/-}$ RD cells by CRISPR-Cas9 mediated targeting. One screen positive clone of *ACBD3*$^{-/-}$ were detected by WB assay. (b) Data shown was the sequence mutations of the positive clone of *ACBD3*$^{-/-}$ used in the study. (c) Effects of ACBD3 on viral entry. RD cells were incubated with EV71 at 37 ℃ for 0.5, 1 and 4h. After washing three times with PBS, the EV71 mRNA was detected by using RT-PCR.

Supplementary Figure S3. 3A interacts with the C-terminal region of ACBD3. (a) Lysates from 293T cells transfect with construct expressing GFP-3A of EV71 were pulled down by different domains of ACBD3 fused to GST or GST alone. (b) Schematic diagram of different constructs of ACBD3 variants. (c) 293T cells were transfected with ACBD3 variants as indicated alone with plasmid expressing GFP-3A. At 24 h after transfection, lysates were immunoprecipitated with antibody against Flag. Samples were subjected to Western blot analysis.

Supplementary Figure S4. The localization of GBF1 and ARF1 after EV71 infection. (a and b) RD were mock infected or infected with EV71 at MOI=5 of PFU/cell. At different time points, cells were fixed and labeled with anti-3A (Green) and anti-GBF1 (Red) (a) or anti-ARF1 (Red) (b) antibodies.

Supplementary Table 1. The primers for 3A variants.

Numbers	Primers
3A 1-30 forward	CTTGCTAGTGTAGATAGTTAAGACGTCGACGGTACCGCGGGCC
3A 1-30 reverse	GGTACCGTCGACGTCTTAACTATCTACACTAGCAAGGAGATCG
3A 1-40 forward	CAGTACTGCAGGGATCAATAAGACGTCGACGGTACCGCGGGCC
3A 1-40 reverse	GGTACCGTCGACGTCTTATTGATCCCTGCAGTACTGGCGCACT
3A 1-50 forward	CCTGAAACTCCCACCAACTAAGACGTCGACGGTACCGCGGGCC
3A 1-50 reverse	GGTACCGTCGACGTCTTAGTTGGTGGGAGTTTCAGGAATGATC
3A 1-60 forward	CTTAATAGAGCAGTGCTTTAAGACGTCGACGGTACCGCGGGCC
3A 1-60 reverse	GGTACCGTCGACGTCTTAAAGCACTGCTCTATTAAGGTGCCGC
3A 21-86 forward	TCTCGAGCTCAAGCTTCGAGCGATCTCCTTGCTAGTGTAGATA
3A 21-86 reverse	ACTAGCAAGGAGATCGCTCGAAGCTTGAGCTCGAGATCTGAGT
3A 41-86 forward	TCTCGAGCTCAAGCTTCGTGGATCATTCCTGAAACTCCCACCA
3A 41-86 reverse	AGTTTCAGGAATGATCCACGAAGCTTGAGCTCGAGATCTGAGT
3A 61-86 forward	TCTCGAGCTCAAGCTTCGATGCAATCTATCGCTACAGTAGTGG
3A 61-86 reverse	TGTAGCGATAGATTGCATCGAAGCTTGAGCTCGAGATCTGAGT
L12I forward :	AGGCCAATTAGGATTAGTATTGAAGAAAAACCAGCCCCAGACGCTA
L12I reverse :	TGGGGCTGGTTTTTCTTCAATACTAATCCTAATTGGCCTGAACTTG
H54Y forward :	CCCACCAATGTAGAGCGGTACCTTAATAGAGCGGTGCTTGTCATGC
H54Y reverse :	AAGCACCGCTCTATTAAGGTACCGCTCTACATTGGTGGGAGCTTCA
I44A forward :	AGGGATCAAGGCTGGATTGCTCCTGAAGCTCCCACCAATGTAGAGC
I44A reverse :	ATTGGTGGGAGCTTCAGGAGCAATCCAGCCTTGATCCCTGCAGTAC

Supplementary figure S1

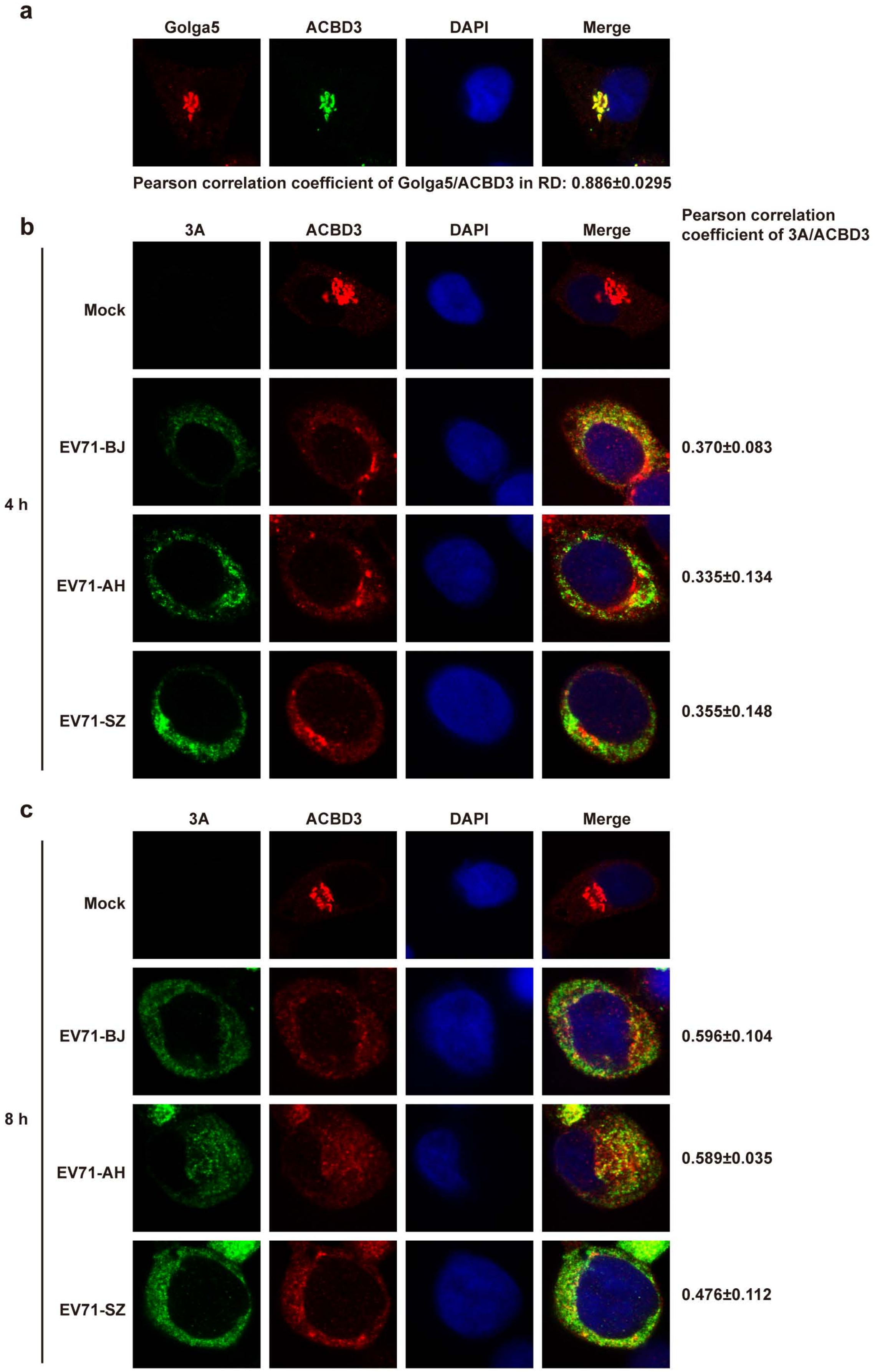

Supplementary figure S2

a

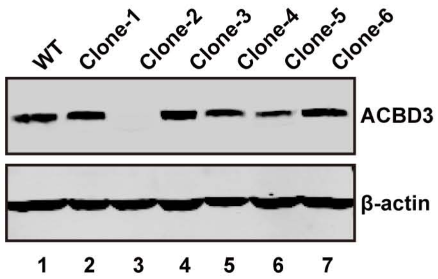

b

```
 Human    P45 G   S   G   R   G   P   G   A   S   G   E   Q   P   E   P
 ACBD3    CCT GGA TCC GGT CGC GGC CCG GGC GCC TCA GGG GAG CAG CCC GAG CCC
Clone-2   CCT GGA TCC GG- --- --- --- --- --- --- --- --- -AT CCC GAG CCC
```

c

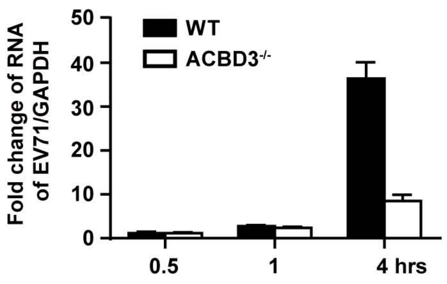

Supplementary figure S3

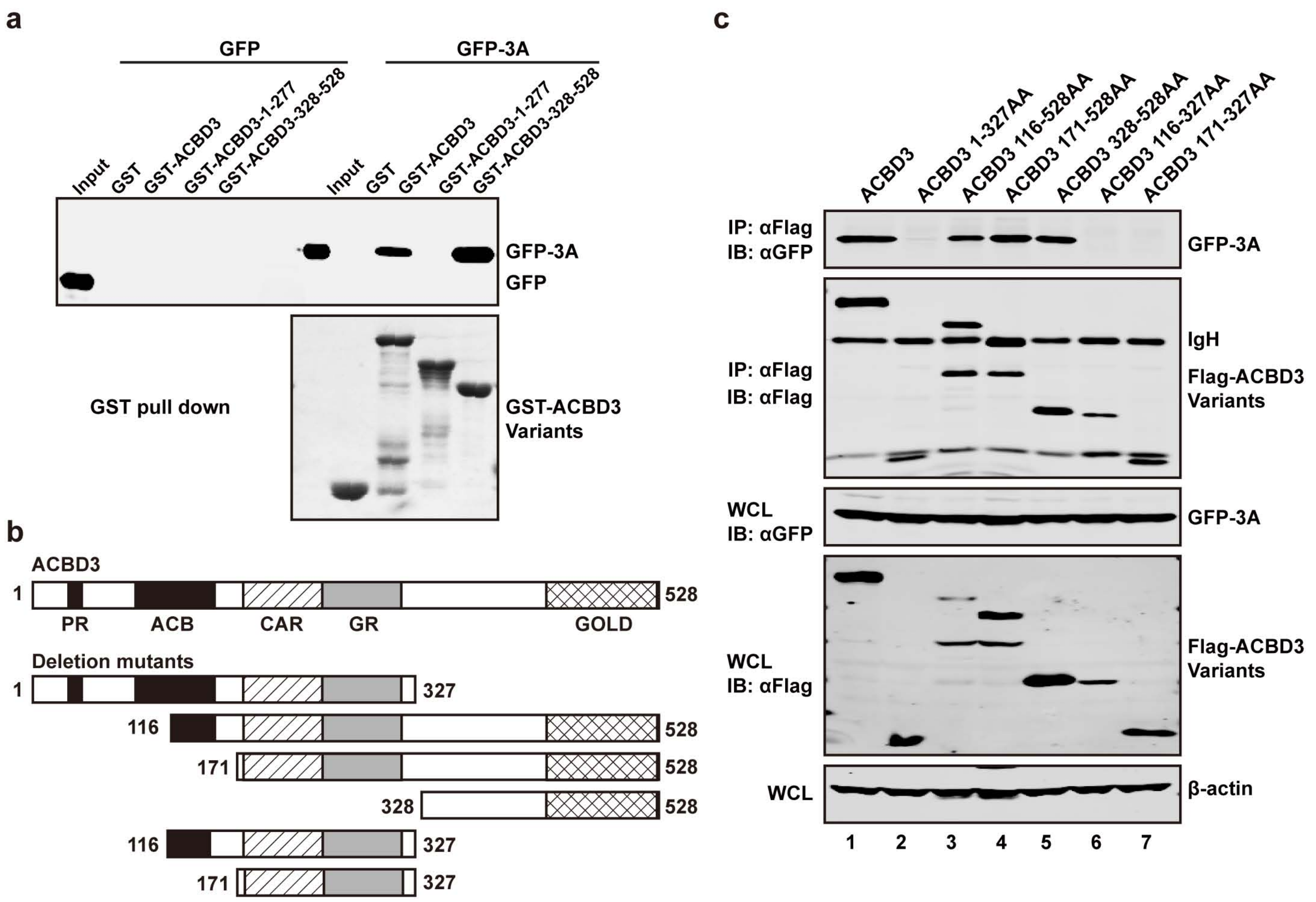

Supplementary figure S4

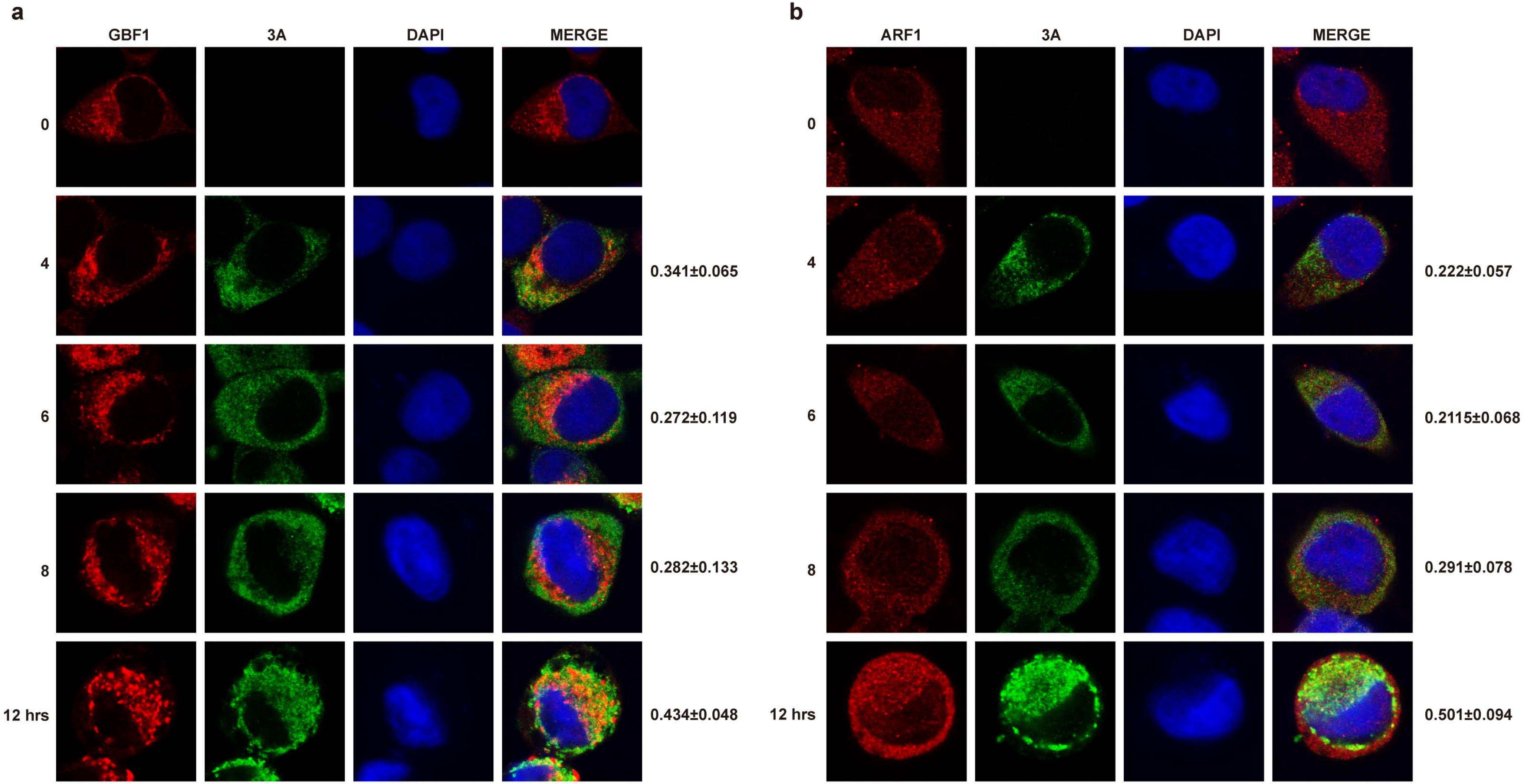

Supplement: Supplementary Information [file srep44592-s1.pdf]
